# Peer Review of “A Machine Learning Explanation of the Pathogen-Immune Relationship of SARS-CoV-2 (COVID-19), and a Model to Predict Immunity and Therapeutic Opportunity: A Comparative Effectiveness Research Study”

**DOI:** 10.2196/24747

**Published:** 2020-10-19

**Authors:** Andy Chang

**Affiliations:** 1 School of Professional Studies Northwestern University Chicago, IL United States

**Keywords:** infectious disease, SARS-CoV-2, COVID-19, public health, immunity: vaccinations, therapeutics, stem-cell growth factor-beta

*This is a peer review submitted for the paper “A Machine Learning Explanation of the Pathogen-Immune Relationship of SARS-CoV-2 (COVID-19), and a Model to Predict Immunity and Therapeutic Opportunity: A Comparative Effectiveness Research Study.”*

## Comments for Authors/Editors

Eric, very clear and concise paper. Very minor suggestions/clarifications/edits only on the abstract, none that I would consider to be substantive.

1. Stylistically, and I went back and forth on this multiple times as I did each of my reviews, the abstract almost reads like it was written by a different person. Your “main” paper flowed beautifully well in what I’ve come to expect from you: you set the stage, you provide the necessary background, you explain succinctly what you did, and then you go into discussions about the potential implications of the work. The abstract almost reads like separate bullets in a white paper, and I realize that you’re going up against wording or space constraints so in the big picture and with resource management, this isn’t one of those tasks or comments that rises to the top.
2. In the conclusion portion of the abstract, do you think it would add value to call out or hat tip the following: (a) any of the limitations that you highlighted (maybe the top one or two?) and (b) the tie-in to economic and socioeconomic factors that you mentioned in the conclusion of the main paper? Again, I recognize that you may be going up against space and word constraints, so this may not be possible.

